# Sex matters: mechanistic insights into sex-driven patterns of autoimmunity and implications for pharmacotherapy

**DOI:** 10.1007/s00210-026-04990-x

**Published:** 2026-01-23

**Authors:** Mariana Soares Guedes, Daniela Yildiz

**Affiliations:** Molecular Pharmacology, Center for Molecular Signaling (PZMS), Center for Gender-Specific Biology and Medicine (CGBM), Pharma Science Hub (PSH), University of Saarland, Saarbrücken, Germany

**Keywords:** autoimmune, sex-bias, pathomechanisms, pharmacotherapy

## Abstract

Autoimmune diseases (AiDs) affect up to 10% of the global population and exhibit striking differences between sexes. These disparities encompass prevalence, incidence, age at onset, disease severity and how patients respond to treatment. This review provides a comprehensive overview of the key biological mechanisms underlying sex-biased autoimmunity across multiple levels, including the immunomodulatory roles of sex hormones, sex-specific innate and adaptive immune responses, X-chromosome gene dosage and escape from inactivation as well as epigenetic regulation of immune pathways. In addition, we address how environmental and lifestyle factors, such as smoking, infections and the gut microbiome interact with sex-specific biology to shape autoimmune risk. Finally, we consider the pharmacological implications of sex differences, including variability in drug efficacy, safety and immune-related adverse events, further highlighting current gaps in sex-stratified clinical research. Recognizing sex as a fundamental biological variable is essential for advancing the understanding of AiDs and for the development of more effective, tailored therapeutic strategies.

## Summary

The term “sex” as a biological construct entails a complex array of biological characteristics, represented by the basic organization of chromosomes, gametes, and reproductive organs, as well as circulating sex steroid hormone concentrations (Short et al. 2013; Nielsen et al. 2021). Similarities between sexes are reflected in the hypothalamic–pituitary structure, hormonal feedback, and gonadotropin-releasing hormone (GnRH) pulsatility, while the differences are reflected in gonads (ovary and testes), sex steroids (estrogen and testosterone), the number of gametes produced (oocyte and sperms), and duration (periodical and continuous process) (Sheng et al. 2021). Gender, on the other hand, is a multifaceted construct including social behaviors and cultural expectations mirroring gender distinctions, which may play a role in disease susceptibility and its progression (Ryan and Mills 2022). One particular aspect of the sex–disease interconnectivity is the vast differences observed in the pathophysiology of certain conditions such as autoimmune disorders (AiDs), where a higher prevalence is observed in women compared to men (Cao et al. 2023). Autoimmune diseases are a varied group of disorders in which the immune system attacks the body’s own tissues, leading to chronic inflammation, tissue damage, and often multisystem involvement. While many individual autoimmune diseases are relatively rare, collectively they represent a substantial global health burden. The underlying mechanisms contributing to these differences are intricate and diverse, so a comprehensive understanding of these processes is imperative for the development of gender-specific therapies and to enhance the prognostic outcomes for individuals afflicted with AiDs. This review will give an overview of the prevalence and incidence of AiDs between men and women, describe the proposed key mechanisms involved in the pathogenesis of AiDs, and address current research efforts, caveats, and future directions towards personalized medicine. Henceforth, the terms female/women as well as men/male will be used interchangeably throughout the text, always referring to the biological sexual differences (male XY and female XX) and not to the way how people may identify (‘gender”).

## Global prevalence, incidence, and distribution of AiDs

**Fig. 1:**
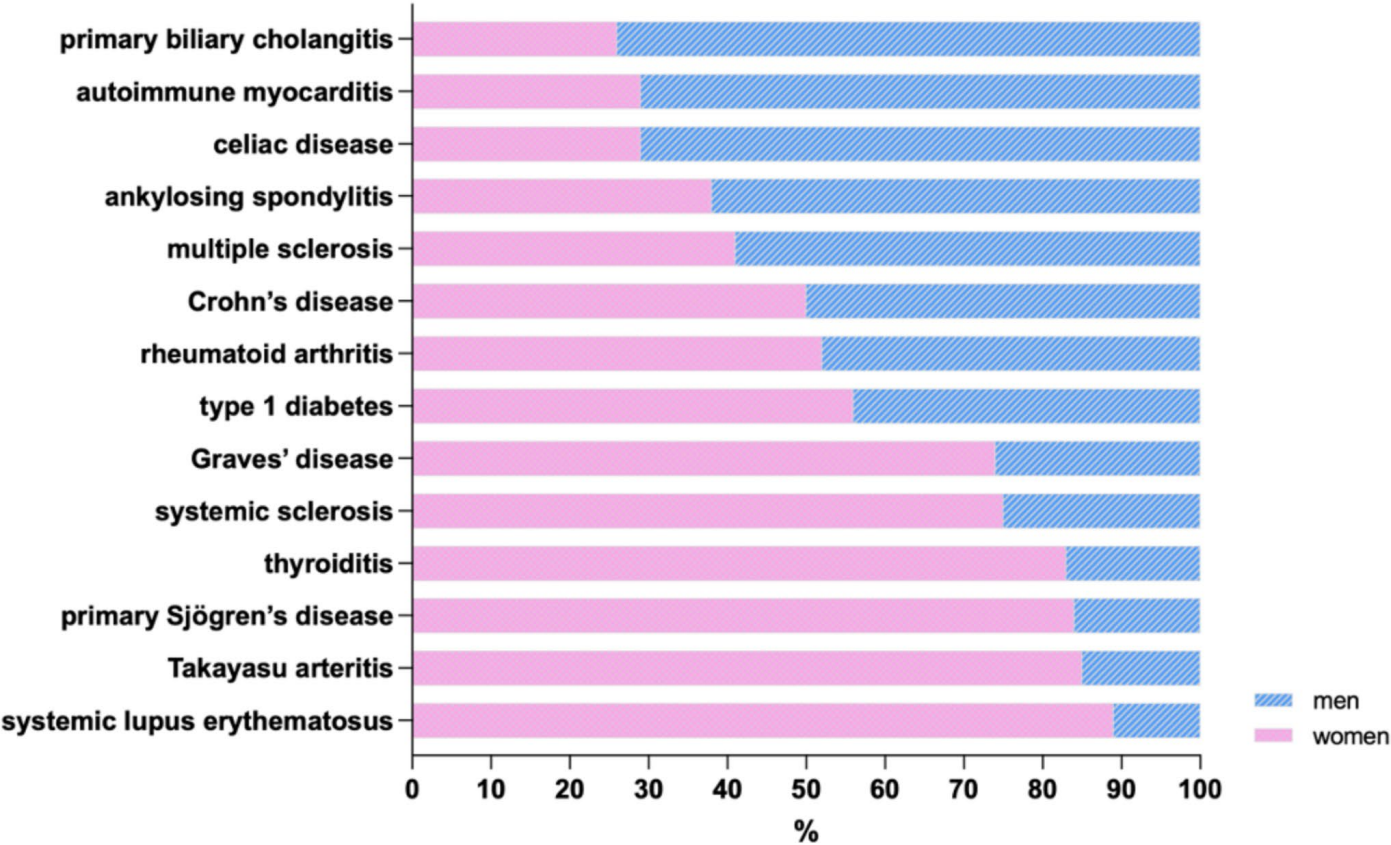
Sex differences in autoimmune diseases between biological sexes. The graph presents the percent difference in prevalence of fourteen autoimmune diseases between women and men, collected from published epidemiologic reports and systematic reviews (Gleicher and Barad 2007; Fairweather et al. 2024). % women/men ratios: type 1 diabetes: 56/44; Crohn’s disease: 50/50; ankylosing spondylitis: 38/62; autoimmune myocarditis: 29/71; primary biliary cholangitis: 26/74; celiac disease: 29/71; multiple sclerosis: 41/59; rheumatoid arthritis: 52/48; Grave’s disease: 74/26; systemic sclerosis: 75/25; thyroiditis: 83/17; primary Sjögren’s disease: 84/16; Takayasu arteritis: 85/15; and systemic lupus erythematosus: 89/11

Autoimmune diseases impact approximately 10% of the population worldwide, with many studies reporting a predominance of women among the patients affected by AiDs (Cooper et al. 2009). For instance, data from China indicate that across multiple autoimmune conditions, women were almost twice as likely to be diagnosed with an AiD than men (Mohamed-Ahmed et al. 2024). This female bias appears not only in prevalence but also in incidence, age at onset, and disease burden (Fig. 1).

Substantial data on global/regional epidemiology, temporal trends, and sex/age patterns has been reported for three prominent AiDs: systemic lupus erythematosus (SLE), rheumatoid arthritis (RA), and multiple sclerosis (MS), with recent reviews re-evaluating the latest numbers of the last five years. The pooled global incidences, assessed per the age standardized incidence rates (ASIR), ranged from 1.4 to 15.13 per 100.000 persons for SLE, from 9.76 to 11.79 per 100.000 persons for RA, and between 0.80 to 0.78 per 100,000 for MS (Tian et al. 2023; Shi et al. 2023; Liu et al. 2025). In terms of prevalence, age standardized prevalence rates (ASPR) worldwide currently estimate 43.7 per 100,000 persons to be affected by SLE, 208.9 per 100,000 persons for RA**,** and 16.05 per 100,000 persons for MS (Tian et al. 2023; Shi et al. 2023; Zhao et al. 2024). Regionally, prevalence and incidence vary widely, with Poland, Barbados, and the USA showing the highest estimates of SLE incidence and the United Arab Emirates, Barbados, and Brazil having the highest prevalence (Tian et al. 2023). Highest ASPR and ASIR for RA are found in Andean Latin America, Central Latin America, and Australasia, while regions like Oceania, Western Sub-Saharan Africa, and Southeast Asia show the lowest rates (Ma et al. 2025). It is clear that trends over the last decades show a growing global burden of at least some AiDs, especially RA, whereas for others (e.g., MS, in global summary), prevalence may be stable or even declining (Cao et al. 2023). However, the female predominance remains a consistent thread across many AiDs. For SLE, global data show that women have incidence and prevalence many times higher than those in men (ASIR: 8.82 women vs. 1.53 per 100.000 persons/ASPR: 78.7 in women vs. 9.26 per 100,000 persons in men) (Barber et al. 2021; Tian et al. 2023). RA women outnumber men worldwide (predicted ASIR for the 2019–2030 period of 18.23 in females and approximately 8.34 per 100,000 people in males), and projections expect this trend to persist (Cai et al. 2023; Ma et al. 2025). For MS, although sex-specific incidence/prevalence rates vary by region and age group, large-scale data from global burden studies and regional registries repeatedly report higher rates in women (data: ASIR 1.03 in females to 0.52 per 100,000 people in males, with ASPR 35.94 in females vs. 14.81 per 100,000 per people in males) (Global Burden of Disease Collaborative Network 2025). Such pronounced sex differences imply that sex (or sex-associated factors) are among the most powerful determinants of AiD risk and burden. As an example, higher estrogen levels in premenopausal women provide an atheroprotective effect, resulting in lower incidence of myocarditis in women compared to men (Di Florio et al. 2020; Barcena et al. 2021).

**Fig. 2:**
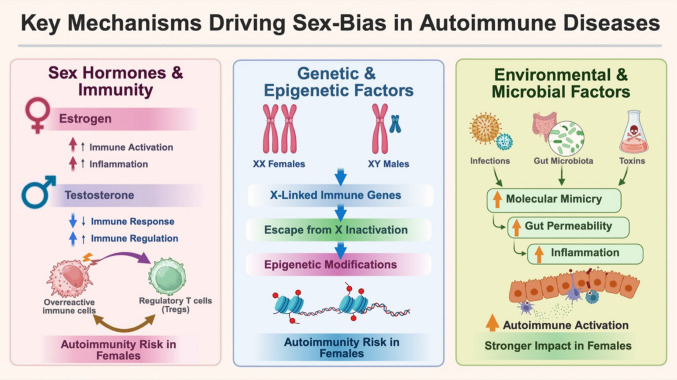
Sex-specific biological mechanisms contributing to autoimmune disease susceptibility. This schematic illustrates three major categories of mechanisms that drive sex differences in autoimmune disease risk. Sex hormones and immune balance: estrogen enhances immune activation and proinflammatory signaling, whereas testosterone suppresses immune responses and supports regulatory pathways. These hormonal effects shift the balance between overactive immune effector cells and regulatory T cells (Tregs), promoting a net increase in autoimmunity risk in females. Genetic and epigenetic factors: females possess two X chromosomes, leading to increased expression of X-linked immune genes and partial escape from X-chromosome inactivation. Together with sex-specific epigenetic modifications, these genetic mechanisms reduce immune regulation and enhance immune reactivity. Environmental and microbial influences: sex differences in exposure to infections, gut microbiota composition, and environmental toxins contribute to molecular mimicry, increased gut permeability, and heightened inflammatory signaling. These factors collectively amplify autoimmune activation and have a disproportionately stronger impact in females. (Created in https://BioRender.com)

Although hormonal changes manifest in a sex-specific manner during adolescence, sex differences in certain AiDs manifest during earlier childhood, a period when hormonal influences are not sex-dependent, suggesting the existence of non-hormonal factors that contribute to these disparities. For AiDs that are more prevalent among children, the sex differences tend to be relatively less skewed towards girls or exhibit greater variability. Childhood-onset SLE shows a marked predominance in young girls, with a female-to-male ratio of 3:1 to 5:1, which increases to 10:1 to 15:1 in adults, indicating a stronger sex bias as age progresses (Valenzuela-Almada et al. 2022). At the same time, male patients with pediatric SLE often experience more severe disease manifestations, particularly in terms of renal and neurological involvement (Chaudhary et al. 2019). Conversely, Kawasaki disease presents a higher incidence in boys compared to girls (approximately 1.5:1 ratio), a trend that reverts in adults (Schnautz and Leggett 2008; Watelle et al. 2025). Type 1 Diabetes (T1D), which is generally considered more predominant in boys, has both age and regional differences, with Korean girls displaying a higher incidence than boys (girl-to-boy ratio of 4.93:4.01), while in western countries such as Germany, boys remain the age with the highest incidence (data from to 2022 RKI report: 32.1 boys per 100,000 person-years and girls at 27.8) (Chae et al. 2020; Robert Koch Institute 2023; Baechle et al. 2023; Buchmann et al. 2023). Overall, the epidemiological evidence paints a picture of autoimmunity as a common, and in some cases growing, global health challenge disproportionately affecting women, with wide variation by disease, region, and age. This shows that autoimmunity cannot be understood solely as a biological phenomenon, but as a result of complex interactions between environmental, socioeconomic, and healthcare factors as well (Fig. 2).

## Key mechanisms in autoimmune sex differences

### Sex hormones

Although sex hormones in isolation are not causative agents in autoimmune disorders, changes in hormonal levels may create a conducive environment for other etiological factors (i.e., genetic, epigenetic, and inflammatory) towards disease onset. Sex hormones act on multiple targets in the body, including the central nervous system (CNS), bones, reproductive organs, and the immune system. Estrogen robustly enhances immune reactivity leading to heightened autoimmunity in females, while testosterone tends to suppress immune responses (detailed review in (Moulton 2018)). The levels of sex hormones in both, humans and animal models, have been shown to correlate with the activity of cytokine-secreting cells (Verthelyi and Klinman 2000). Studies show that dehydroepiandrosterone (DHEA) promotes the production of type 1 cytokines (IL-2 and IFN-γ) and the development of cell-mediated immunity, while estrogen stimulates B cell activation, in part by stimulating type 2 cytokines (IL-4, IL-6, and IL-10) (Daynes et al. 1990; Cao et al. 2019). On the other hand, testosterone exerts its immunosuppressive properties by downregulating the production of proinflammatory cytokines such as IL-1β, TNF-α, IL-6, and IL-17 (Corrales et al. 2006; Babaloo et al. 2015). In addition, research on mouse models indicates that lowering testosterone levels stimulates polyclonal B cell activation, leading to an augmented expression of autoantibodies that are characteristic of AiDs (Viselli et al. 1997; Wilhelmson et al. 2018). One prominent example of dysregulated sex hormone levels contributing to the altered cytokine milieu is SLE (Buendía-González and Legorreta-Herrera 2022). Specifically, estrogen accelerates SLE progression in mouse models of lupus that have been subjected to gonadectomy (removal of the sexual organs) or hormone deprivation/supplementation, where estrogen supplementation accelerates disease progression in female mice and in males without gonads, while androgen supplementation improves SLE in female mice (Lahita 2016). In humans, estrogen metabolism has been found to be increased in SLE patients, with higher estrogen receptor (ER) transcripts being expressed in peripheral blood mononuclear cells and T cells, with a linked increase in active ER proteins, despite no actual differences in plasma estrogen levels being found between healthy vs. SLE women (Suenaga et al. 2001; Kassi et al. 2001; Moulton and Tsokos 2015).

Three important hormonal transformations in a woman’s life occur during puberty, pregnancy, and menopause. The increase in estrogens and testosterone during puberty has a profound impact on immune and metabolic regulation, significantly increasing the risk of autoimmune diseases in girls (Resztak et al. 2023). One example is Grave’s disease, a peculiar AiD, since it does not lead to target tissue destruction but instead involves hyperplasia of the thyroid gland and upregulated thyroid functions, characterized by increased serum levels of thyroid hormone (TH), depleted levels of thyroid-stimulating hormone (TSH), and detectable levels of TSH receptor antibodies (Smith and Hegedüs 2016). Recently, it was shown that women have higher B cell-activating factor protein (BAFF) transcripts and protein levels than men and that administering estradiol to male mice in a spontaneous autoimmune thyroiditis model leads to an increase in BAFF levels, halting disease progression (Cheng et al. 2021). This relationship suggests that the fluctuations in estrogen levels associated with puberty in women could be a significant factor underlying the elevated incidence of AiDs among females. RA and Sjogren’s syndrome tend to impact women over the age of 40 more frequently than their male counterparts, a finding that has been hypothesized to be related to the observed decrease in estrogen levels during menopause (Angum et al. 2020). RA primarily affects the joints, typically resulting in warm, swollen, and painful joints, involving most frequently the wrists and hands, with the same joints typically involved on both sides of the body (Talsania and Scofield 2017). In turn, Sjogren’s syndrome is a complex autoimmune disorder resulting from the immune system erroneously directing its attack towards the individual’s own salivary and lacrimal glands, leading to difficulties in swallowing, continuous dry mouth sensation, along with dry and inflamed red eyes (Brandt et al. 2015). Elevated levels of estrogen have been detected in the synovial fluid of patients suffering from RA, which has been attributed to the role of aromatase in peripheral tissues (Cutolo et al. 2006). Inflammatory cytokines like TNFα, IL-1, and IL-6, produced by macrophages, enhance the activity of aromatase, which is responsible for converting androgens (DHEA, testosterone, progesterone) into 17-β-estradiol, which subsequently interacts with immunocompetent cells, thereby activating macrophages, leading to a cycle that promotes the production of proinflammatory cytokines in the synovial joint (Capellino et al. 2008; Holroyd and Edwards 2009). Pregnancy is viewed as protective for RA, given the prototypical rise in estrogen and progesterone levels, with menopause and the post-partum periods being frequently linked to disease exacerbation. Secondary osteoporosis, a condition characterized by reduced bone mass and increased fracture risk, can develop as a consequence of AiDs such as RA and SLE. In these cases, chronic inflammation, immune-mediated bone damage, reduced mobility, and corticosteroid treatments all contribute to bone loss. Osteoimmunology studies reveal that cytokines like TNF-α, IL-1, and IL-6 stimulate osteoclast activity (bone resorption) while impairing osteoblast function (bone formation), accelerating bone loss (Zupan et al. 2013). This means that immune dysregulation can accelerate bone loss, despite the fact that the core disease mechanism in osteoporosis isn’t necessarily autoimmune in origin.

F:M, female: male (approximate typical ratio, where reported); ER, estrogen receptor; DC, dendritic cell; TLR, toll-like receptor; IFN, interferon; miRNA, microRNA; BAFF, B cell-activating factor protein; SNPs, single-nucleotide polymorphisms.

Glucocorticoids possess potent anti-inflammatory properties and are routinely prescribed for managing autoimmune and inflammatory conditions. The impact of sex hormones on glucocorticoid levels occurs through the modulation of the hypothalamic–pituitary–adrenal axis, thereby influencing immune responses (Weiser and Handa 2009). Specifically, estrogens can induce thymic atrophy by eliminating early thymic progenitors and inhibiting the proliferation of thymocytes (Zoller and Kersh 2006). This action can indirectly influence the role of glucocorticoids in T cell development, as they are involved in the selection of T cell receptors in the thymus (Brown and Su 2019). Both estrogens and glucocorticoids bind to nuclear receptors that regulate gene expression; thus, the presence of estrogen receptors in immune cells suggests that estrogens can modulate the expression of genes that are also targets of glucocorticoid action, potentially leading to complex interactions between these hormones at the molecular level (Brown and Su 2019).

Given the broad range of action of sex hormones and their critical role in the physiology of reproductive tissue, bone, cardiovascular, lipid, and immune system, the modulation of sex hormones or their receptors has been implemented in therapies for cancer, bone diseases, and AiDs. A comprehensive understanding of the physiological interplay between sex hormones and immune function, as well as its potential pathological ramifications, has been yielding significant insights into AiDs and shed light on new therapeutic interventions.

### Immune system differences

Sex differences in immunity are well-documented, with women generally exhibiting stronger innate immune responses and men’s immune systems often showing stronger adaptive responses (Ryan and Mills 2022). Over the last decades, many publications have addressed the action of sex hormones on T and B lymphocytes, yet only a few studies focused on sex hormones’ impact on innate immune cells (Klein 2012). Female antigen-presenting cells (APCs) appear to be more effective at stimulating T cells than male APCs, with estradiol enhancing dendritic cell maturation (Wira et al. 2000). In humans, gene expression microarray analysis comparing the expression pattern of T cells after stimulation in healthy men and women identified an overexpression of immune response genes with inflammatory and cytotoxic functions in women (i.e., interferon-gamma (IFN-g), lymphotoxin b (LTb), granzyme A (GZMA), interleukin-12 receptor b2 (IL12Rb2), and granulysin (GNLY)) (Hewagama et al. 2009). Moreover, the presence of estrogen response elements in the promoters of half the overexpressed immune genes was identified, whereas only 10% of the genes were male-biased genes, directly linking estrogen response to immune sexual dimorphism and AiD exacerbation (Hewagama et al. 2009). Additionally, Th1 and Th17 cells (two proinflammatory immune cell types highly implicated in MS pathology) exhibit sex differences within their responses as well (Damsker et al. 2010). In the experimental autoimmune encephalomyelitis (EAE) mouse model (representative of the human multiple sclerosis phenotype), it has been shown that treatment with estradiol at levels similar to those observed during pregnancy not only suppressed IL-17 production but increased the frequency of regulatory T cells (Tregs) (Wang et al. 2009). Tregs play a crucial role in regulating inflammation by secreting immunosuppressive cytokines such as IL-10 and TGF-β, and dysfunctions in Treg cell activity, which result in unchecked autoreactive T cells, are frequently observed in AiDs (Engler et al. 2017). Progesterone has been shown to interact with the glucocorticoid receptor on T cells, increasing Treg cell frequency and ultimately suppressing AiD disease activity in pregnant female mice that were undergoing EAE (Lee et al. 2011; Engler et al. 2017). Similarly, in humans, pregnancy induces changes in the maternal immune system in order to protect the fetus, and pregnant women display enhanced immunoglobulin and autoantibodies production as well as a shift from Th1 response to a type Th2, linked to the increased progesterone levels during gestation (Marzi et al. 2003). Taken together, these data indicate that targeting sex hormones can be a potential avenue to tackle immune exacerbations in AiDs, at the very least in the case of multiple sclerosis. The EAE model has also shown that there is a male-specific protective effect of innate lymphoid cells 2 (ILC2), an important source of IL-5, IL-9, and IL-13 that drive Th2, anti-inflammatory responses (Russi et al. 2015; Kadel et al. 2018). Further research on human T cells has demonstrated that testosterone itself can stimulate the expression of Foxp3 in Tregs through direct engagement with an androgen receptor binding site within the Foxp3 locus, resulting in epigenetic modifications that facilitate the differentiation of Treg cells (Walecki et al. 2015).

The recent COVID-19 pandemic has revealed differences in immune responses between males and females, with men developing more severe COVID-19 symptoms and having a higher risk of mortality compared with women, and women show higher levels of activated T cells during SARS-CoV-2 infection (Takahashi et al. 2020). Yet, the degree of sex differences in T helper cell and Treg cell functionality remains poorly defined, and additional research into the frequency and functionality of Treg cell populations, as well as the expression of immune checkpoints in both females and males, is necessary. Generally, females show greater humoral immunity than men, which is related to higher B cell counts and stronger antibody responses than males (Fink and Klein 2018). B cells express both estrogen receptor-α and receptor-β, and estradiol promotes the survival and activation of immature B cells, resulting in the development of mature reactive B cells (Santana-Sánchez et al. 2024). This phenomenon has been linked, at least partially, to the higher incidence of SLE in women (Grimaldi et al. 2002). Additionally, a multitude of mechanisms seems to be implicated in the disruption of immunological tolerance and the increase of plasma cell activity, reduction of the bone marrow and thymic mass, the emergence of extramedullary hematopoietic sites, and a modified susceptibility of B cells to apoptosis (reviewed in (Buendía-González and Legorreta-Herrera 2022b)). Sex differences in the susceptibility to and progression of AiDs may be a reflex of the sexual dimorphism found in the balance between proinflammatory Th1, Th17, and B cells versus protective Th2 and Tregs. However, there is still limited data from patient studies linking these in vitro and animal-derived findings to the immune sex differences in AiDs observed between both sexes. More comprehensive clinical trials on sex differences observed in the response to novel therapeutics that target IL-17/Th17 cells or B cells should provide more information to address current knowledge gaps.

### Genetic and epigenetic factors

The human X chromosome encodes approximately 2000 genes, a substantial number of which are implicated in the regulation of immune functions (Ross et al. 2005). This chromosome harbors genes for pattern recognition receptors (PRRs), cytokine receptors, and transcription factors (Balaton et al. 2018). In females, one of the X chromosomes is typically inactivated to balance gene expression with males, who have only one X chromosome. It is estimated that around 15% of genes evade X-inactivation in humans, resulting in elevated gene expression levels in females compared to their male counterparts, namely, genes involved in the Toll-Like receptor 7 (TLR7) signaling pathway, which have been implicated in increased autoantibody production and inflammation in women (Wang et al. 2016). The "four core genotype" (FCG) mouse model has proven instrumental in delineating the phenotypic outcomes attributable to sex chromosomes versus gonadal hormones (Arnold 2020). This model generates XX and XY mice with testes as well as XX and XY mice with ovaries by manipulating the presence of the SRY testis-determining gene. A phenotypic variation observed between XX and XY mice exhibiting identical gonadal types would therefore arise from the influence of sex chromosomes. In contrast, a phenotypic distinction between mice possessing the same sex chromosome composition yet differing in gonadal type would result from the impact of gonadal hormones (Burgoyne and Arnold 2016; Mauvais-Jarvis et al. 2017). This differentiation is critical, as disparities in immune function can emerge from both the complement of sex chromosomes and the levels of sex hormones. Recently, it was demonstrated that altering the inactivation of the X chromosome in XX female mice led to a spontaneous development of inflammatory signs of SLE, such as antinucleic acid autoantibodies and expansion of monocytes, macrophages, and dendritic cells as a result of dysregulation of TLR7 signaling in macrophages, an observation that was not replicated in XY female and XY male mice (Huret et al. 2024).

Certain human leukocyte antigen (HLA) class II alleles, such as HLA-DRB1*15:01 and HLA-DQB1*03:02, are associated with a higher risk of MS (Moutsianas et al. 2015). These alleles are part of the major histocompatibility complex (MHC) system, also known as the human leukocyte antigen (HLA) system, which encodes cell surface molecules specialized in presenting antigenic peptides to T cells that help to differentiate between self and nonself (Cruz-Tapias et al. 2013). While the exact mechanisms are not yet fully understood, studies suggest that the expression of these HLA alleles predisposes women to a higher susceptibility to develop MS as a result of the epigenetic modulation induced by cigarette smoking, an important lifestyle factor discussed in more detail in the “Environment, lifestyle, and the microbiome” section (Klein and Flanagan 2016).

Epigenetic regulation also plays a significant role in modulating the type I interferon (IFN) pathway, which is crucial in the pathogenesis of SLE (Mazzone et al. 2019).The methylation status of genes involved in the IFN pathway can influence their expression. The IFI44L gene promoter is found to be hypomethylated in SLE patients, which is in turn associated with increased expression of IFN-stimulated genes (ISGs) (Liu et al. 2020). Additionally, the X chromosome contains numerous microRNAs (miRNAs) that contribute to the modulation of immune responses (Selmi et al. 2012). Abnormal expression of miR-146a found in SLE patients has been found to interfere with type I-IFN expression in a feedback-loop manner, and reduced levels of lncRNA MALAT1 in SLE are linked to the inhibition of IRF3, a key downstream factor in the IFN pathway (Qu et al. 2015; Liu et al. 2020; Hou et al. 2021). Furthermore, in SLE, abnormal DNA patterns are observed in genes like CD11a, CD40L, and CD70, which are associated with immune cell activity and disease susceptibility, while for RA, histone modifications in synovial fibroblasts lead to increased expression of inflammatory cytokines such as TNF-α, contributing to disease progression (Gaur et al. 2016; Karouzakis et al. 2018). Other factors such as epigenetic enzymes, which mediate histone methylation, are found to be upregulated in CD4 + T cells in lupus patients, increasing T-cell adhesion to endothelial cells, while inhibitors targeting these enzymes have been shown to have great potential as therapeutic targets (Tsou et al. 2018). Thus, the association between dysregulated immune cells and signaling pathways with epigenetics has shown great potential as both diagnostic and prognostic biomarkers in AiDs (Table 1). However, to date, the severe adverse effects observed by drugs targeting these markers have limited their translation to clinical applications and treatment options for patients with SLE and RA (Mazzone et al. 2019; Hogg et al. 2020).

**Table 1:** Summary of the key mechanisms implicated in sex bias in the most prevalent reported autoimmune diseases in humans

Disease *(typical sex bias)*	Key sex-bias mechanisms	References *(mechanism-focused)*
**Systemic lupus erythematosus (SLE)** (≈ ~ **9–14:1 F:M**)	X-chromosome gene dosage/escape from X-inactivation (i.e.: TLR7 overexpression) Estrogen/ER signaling boosting B cell & DC activity and TLR signaling Higher type I IFN signature in females Epigenetic/miRNA regulation (i.e., DNA hypomethylation of CD40L on X chromosome)	(Palmer et al. 1993; Grimaldi 2006; Dolff et al. 2011; Farivar and Aghamaleki 2018; Ma et al. 2023; Charras et al. 2025)
**Sjögren’s syndrome (primary SS)** (≈ ~ **6:1 F:M**, variable)	X-chromosome dosage/escape Estrogen effects on glandular immunity and autoantibody formation B cell/BAFF overactivity in females Epigenetic dysregulation in salivary glands (i.e., hypomethylation and overexpression of LINE-1)	(Mavragani et al. 2016; Imgenberg-Kreuz et al. 2018; Punnanitinont and Kramer 2023; Zhang et al. 2023; Xuan et al. 2024)
**Primary biliary cholangitis (PBC)** (≈ ~ **4–10:1 F:M**)	Sex hormones (estrogen) and epigenetic regulation (i.e., hypermethylation of FUNDC2) X chromosome contributions Sex differences in bile-acid and immune signaling Environmental/epigenetic triggers (i.e., upregulated miR-506) interact with female-biased immunity	(Smyk et al. 2012; Gerussi et al. 2018; Rodrigues et al. 2018; Ismail et al. 2023; Ma et al. 2024; Ronca et al. 2025)
**Rheumatoid arthritis (RA)** (≈ ~ **2–3:1 F:M**)	Sex hormones influence B-cell and T-cell function Androgen deficiency may increase risk/severity in men Epigenetic and X-linked effects (i.e., SNPs in HLA genes encoded in the X-chromosome)	(Libert et al. 2010; Raine and Giles 2022; Cheng and Wang 2023; Huang et al. 2025)
**Multiple sclerosis (MS)** (≈ ~ **1.7–3:1 F:M**)	Female-predominant adaptive immune activation Pregnancy-associated immunomodulation (pregnancy → decreased relapse) Neurosteroid and androgen neuroprotection in males Sex hormones modulate inflammation and repair	(Knudsen 2009; Patas et al. 2013; McCombe 2018; D’Anca et al. 2023)
**Autoimmune thyroid disease (Hashimoto’s, Graves’)** (≈ ~ **4–6:1 F:M**)	Estrogen receptor effects on thyroid autoimmunity X-linked susceptibility loci Altered B cell/autoantibody regulation Epigenetic modulation of thyroid antigen presentation (i.e., skewed X chromosome inactivation, aberrant DNA methylation of FOXP3, CTLA-4 genes; dysregulation of miR-146a and miR-155)	(Limbach et al. 2016; Wang et al. 2017; Cheng et al. 2021; Xie et al. 2025; Buczyńska et al. 2025)
**Type 1 diabetes (T1D)** (≈ ~ **1:1.8 F:M**) *(sex bias variable by age/region; modest overall)*	Sex differences in immune development (i.e., estrogen-responsive enhancers at *IL2RA* modulate Treg stability, with estrogen-driven *FOXP3* induction enhancing tolerance mechanisms in women) Puberty/hormonal fluctuation (i.e.: insulin autoantibodies at the onset of type 1 diabetes is higher in males than females during adolescence) Gut microbiome may influence age-dependent risk (i.e., male bias in some pediatric cohorts, female bias in some adult cohorts)	(Williams et al. 2003; Plamper et al. 2017; Codner et al. 2020; Mousavi et al. 2020; Minniakhmetov et al. 2024; Qu and Hakonarson 2025)
**Myasthenia gravis** *(early-onset female predominance; late-onset more male)*	Sex-dependent thymic pathology (i.e., higher IFN response in women drives thymic inflammation and follicular thymic hyperplasia; estrogen influences tissue restricted antigens presented to the thymus, tilting central tolerance towards higher risk of self-reactivity in women) Hormonal modulation of autoantibody production (i.e., estrogen downregulates autoimmune regulator (AIRE) expression in thymic epithelial cells, via estrogen receptor α and increased CpG methylation in the AIRE promoter, impairing negative selection of autoreactive T cells); Age interacts with sex hormones to shift bias (i.e., early-onset more frequent in females younger than 40)	(Nancy and Berrih-Aknin 2005; Berrih-Aknin and Le Panse 2014; Dragin et al. 2016; Vinciguerra et al. 2023; Myllynen et al. 2025)
**Autoimmune myocarditis** (≈ ~ **1:3.5 F:M**) *(predominant male bias)*	Androgen-related enhancement of certain innate responses (e.g., proinflammatory macrophage/Th17 pathways) Sex-differential viral responses (i.e., in mouse models of CVB3 myocarditis, male mice exhibit higher TLR4 expression promoting exaggerated Th1 proinflammatory response and suppression of Tregs, driving more severe myocarditis phenotype)	(Roberts et al. 2012; Nguyen and Wu 2022; Liu and Han 2024; Thevathasan et al. 2024; Di Florio et al. 2024; Schütze et al. 2025)

### Environment, lifestyle, and the microbiome

Exposure to certain environmental triggers, such as chemicals, drug intake, or contact with infectious agents such as viruses, can interact with genetic and hormonal factors to influence the establishment or progression of AiDs. For instance, cigarette smoking has a pronounced effect on multiple sclerosis risk in individuals possessing certain HLA genotypes: the class II variant HLA‑DRB1*15:01 has a strong association with an increased risk of MS, whereas the class I variant HLA‑A*02 is linked with MS protection (Olsson et al. 2016). Smoking promotes activation of proinflammatory pathways and alters post‑translational modification of peptides, which in turn can lead to the overactivation of resident antigen-specific autoimmune T cells in the central nervous system through recognition peptides presented by HLA‑DRB1*15:01 molecules (Rojas-Villarraga et al. 2013). Elevated levels of cotinine (indicative test of cigarette smoking) in the serum from patients before they developed MS were associated with increased risk of developing MS later in life (Salzer et al. 2013). MS is more prevalent in women, and smoking not only aggravates disease progression, but it contributes to its development in otherwise deemed healthy women (Olsson et al. 2016). Similarly, smoking also increases the risk of RA by 2.4 times in women, and the risk is even higher in men smokers (up to 4.4 times higher) (Krishnan et al. 2003). Mechanistically, when associated with the HLA-DRB1*04 gene variants, smoking may initiate RA by increasing citrullination of proteins in the airway, which produce autoantigens that (when presented to the immune system by the HLA-DRB1 SE alleles) trigger an autoimmune response, producing anticyclic citrullinated antibodies that target the joints, contributing to the establishment of RA (Gregersen et al. 1987; Roh 2019; Hedström et al. 2019).

The gut microbiome plays a critical role in immune regulation, and a bidirectional relationship between sex hormones and gut microbiota composition has recently been found for RA (Yeoh et al. 2013; Rosser et al. 2022). A study using a humanized mice model of RA showed striking differences in gut bacteria abundances, not only between arthritis-susceptible vs. -resistant mice but also between the sexes of each category of mice, resulting in a higher propensity for initiation of RA skewed towards female susceptible mice (Gomez et al. 2012). In humans, new onset RA patients (NORA) tend to have increased *Prevotella copri* and lower levels of Bacteroides in the gut microbiota (Pianta et al. 2017). In China, a study looking into RA patients shows increased levels of *L. salivarius* in the gut and *P. copri* in the gut for the first year after disease onset (Zhang et al. 2015), but sexual dimorphism was not directly implicated in these differences. Dietary patterns and sun exposure have also been implicated in preventing or exacerbating autoimmune disorders. In particular, increasing vitamin D levels and sun exposure before the age of 20 has been associated with a lower risk of MS later in life, which has further been correlated with decreased damage to axons upon vitamin D supplementation (Bjørnevik et al. 2014; Sandberg et al. 2016).

Viral infections are an unexpected environmental factor in certain AiDs, such as autoimmune myocarditis, with higher prevalence in men (Rose 2009). Coxsackieviruses, particularly Coxsackievirus B3 (CVB3), trigger autoimmune myocarditis through several interlinked mechanisms. The dominant pathways involve mitochondrial injury, release of cardiac antigens, complement dysregulation, and immune cross-reactivity (Tam 2006; Esfandiarei and McManus 2008). CVB3 infection damages cardiomyocyte’s mitochondria, leading to the release of mitochondrial and cardiac proteins into the bloodstream. These act as danger signals, activating innate immune receptors (e.g., TLR4 and inflammasome) and promoting the presentation of self-antigens to the immune system (Esfandiarei and McManus 2008). In turn, the release of cardiac antigens (e.g., cardiac troponin, myosin, and SERCA2a) leads to the activation of autoreactive T and B cells, resulting in autoantibody production and cytotoxic T cell-mediated tissue damage (Won et al. 2022). In experimental models of CVB3-induced myocarditis, significant levels of troponin autoantibodies have been detected following the initial release of troponin I, highlighting this link between the viral infection and the development of a cardiac-specific autoimmune response(Latva-Hirvelä et al. 2009). Troponin is the preferred marker in detecting acute coronary syndrome, a severe life-threatening condition (Salaun et al. 2024). The presence of antitroponin autoantibodies in the serum following CVB3-driven myocarditis may cause a false-negative evaluation of troponin levels and delay treatment of acute coronary syndrome (Kaya et al. 2010; Vilela et al. 2017). In relation to sex differences, antitroponin antibodies are usually found in lower levels in women, but are better predictors of follow-up coronary events when elevated, while men tend to present higher basal levels of these antibodies compared to women of the same age range and have higher death rates associated with coronary syndrome (Omland et al. 2015; Kimenai et al. 2021). Thus, the concentrations of autoantibodies against cardiac troponin within the general population serve as a marker for functional and structural changes in the heart tissue, which correlate with increased risk and prevalence of myocardial events in men than in women.

Certain medications, vaccines, and cancer treatments have also been implicated in the development of AiDs. Influenza and smallpox vaccines have showed to increase the incidence of myocarditis and, more recently, the BNT162b2 COVID19 vaccine from BioNTech/Pfizer has also been associated with higher rates of myocarditis or the development of autoimmune neurologic adverse events such as Guillain–Barré syndrome (Engler et al. 2015; Mahroum et al. 2022). Additionally, immune checkpoint inhibitors (ICIs) used for cancer treatment have recently been under scrutiny due to rare, but severe, immune-related side effects (Khan and Gerber 2020). By blocking critical regulators of immune tolerance such as the PD-1, PD-L1, and CTLA-4, ICIs enhance antitumor immunity, but can also disrupt self-tolerance, leading to immune-related adverse events like myocarditis (Zhang et al. 2022). Pathologically, ICI-induced myocarditis is characterized by the infiltration of cytotoxic CD8 + T cells and, in some cases, autoantibody production against cardiac antigens such as α-myosin and troponin T, suggesting both cell-mediated and humoral autoimmunity (Won et al. 2022). The loss of checkpoint-mediated inhibition allows the expansion and activation of autoreactive T cells, which target cardiac tissue and may be further exacerbated by increased T cell receptor diversity and impaired peripheral tolerance mechanisms (Moslehi et al. 2021). Furthermore, men tend to present with more severe inflammation and higher levels of heart failure biomarkers, with most studies reporting a male predominance in ICI-associated myocarditis (between 67 and 77%) (Cozma et al. 2022; Vasbinder et al. 2022).

Despite emerging hypotheses for the development of autoimmune phenomena after environmental exposures, there is no strong epidemiological evidence congruent with causality criteria in the occurrence of gender-differential, and the exact mechanisms accountable for the development of environmentally induced autoimmune disorders remain poorly understood. Nevertheless, experimental models and observational studies have pointed towards this association, but a clear link of these effects on the distinct molecular signaling pathways or immune responses between genders has also not been clearly established and requires more thorough analysis.

## Implications for treatment and future directions

Understanding the mechanisms underlying sex differences in AiDs has several practical implications, particularly in the fields of medicine and public health. Surveillance systems need to be sensitive and comprehensive, public health policy must recognize the chronic and often lifelong burden of these diseases, and research must continue to chart both biological and environmental determinants of autoimmunity across populations. By identifying the role of sex hormones, immune genes, and epigenetic regulators, researchers can develop more targeted therapies for autoimmune diseases by taking these sex-specific factors into consideration. How the environment and genetic predisposition contribute to higher rates of autoimmunity in women can inform preventive strategies, such as lifestyle modifications (i.e., reducing sodium intake, maintaining adequate vitamin D levels, and avoiding smoking) or allow for the development of early interventions that could be designed to mitigate these risks, especially in populations with a high prevalence of AiDs. Moreover, recent reports show a dramatic increase in the prevalence of autoimmune disorders as a result of the last decades’ alterations in food, xenobiotics, air pollution, stress, and climate change, with increasing burden to public healthcare costs (Miller 2023).

Albeit slow to be acknowledged as an important variable in human disease and treatment approaches, significant strides have been taken to understand the mechanisms that contribute to the higher incidence of AiDs in women. The 2015 directive from the NIH mandating the reporting of sex differences in cellular, animal, and human studies has resulted in an increase in such disclosures (National Institutes of Health 2015). A substantial number of earlier publications failed to disclose the sex of the animals or cell types. Importantly, a frequently neglected aspect that influences the interpretation of sex differences in immune responses is the presence or absence of mast cells in various mouse strains. Mouse strains abundant in mast cells, like BALB/c mice, primarily exhibit Th2 immune responses to antigens/autoantigens, whereas strains with fewer mast cells, such as C57BL/6 mice, predominantly show Th1 immune responses (Feyerabend et al. 2016; Sabaté San José and Petersen 2024). Thus, it is essential to document sex differences in mouse models while considering the specific mouse strain. Another critical gap is the absence of recent epidemiological studies that report the prevalence of all the existing AiDs in the United States, or the prevalence of each single AiD along with their corresponding sex ratios. These studies are essential for increasing the understanding of sex differences in autoimmune diseases and for encouraging further research into the underlying mechanisms. Some mechanisms in the different pharmacokinetic response between males and females should also be considered, given that men appear to have greater activity than women, e.g., for cytochrome P450 (CYP) (Soldin and Mattison 2012). Other important aspects are related to physiological differences, such as a generally lower body weight and organ size, a higher percentage of body fat, a lower glomerular filtration rate, and different gastric motility in women compared to men (Meibohm et al. 2012). The existence of gender-related autoimmune mechanisms may represent a therapeutic approach that requires further attention.

Several early-phase clinical trials are currently underway focusing on genetic therapy approaches such as DNA vaccines, viral vectors, and stem cells for AiDs with high prevalence in women, i.e.: RA (Evans et al. 2005), MS(Bar-Or et al. 2007), and SLE (Dao et al. 2024). FT819, an iPSC allogenic CAR-T designed against CD19 for the depletion of pathogenic B cells in SLE patients, is currently in a Phase I clinical trial (NCT06308978), and a combination strategy of Alemtuzumab with CD314 stem cell transplantation is seeking to improve clinical symptoms in patients with severe Crohn’s disease, particularly those with refractory intestinal fistulas (NCT00692939). Yet, none of the trials so far have analyzed sex as a variable for treatment efficiency and/or improvement of disease prognosis. Despite the growing social awareness of transgender people, they remain largely underrepresented in the scientific literature (Schulz 2018). With the clear association of sex hormones in the modulation of the immune system, it is imperative to understand the long-term health outcomes in transgender people receiving gender-affirming hormone therapy (GAHT) (Yalcinkaya et al. 2024; Lakshmikanth et al. 2024). Direct experimental data from a recent study performing longitudinal systems-level analyses in 23 trans men showed that masculinizing testosterone treatment attenuates IFN-I responses in plasmacytoid dendritic cells and monocytes while enhancing some TNF/IL-6 pathways, with GAHT inducing reproducible shifts in immune signaling that mirror sex differences seen in cis populations (Lakshmikanth et al. 2024; Nguyen et al. 2025). While clinical evidence to date does not reveal major population-level surges in autoimmune disease incidence upon GAHT (White et al. 2022; Wiepjes and Heijer 2023), few scattered case reports reveal new onset autoimmune disease profiles associated with feminizing therapy for SLE (Chan and Mok 2013; Campochiaro et al. 2018) and an amelioration of clinical disease course of primary biliary cholangitis on a trans man after commencement of testosterone therapy (Henze et al. 2025). Thus, bringing awareness to the medical and research communities to better represent, protect, and advise vulnerable individuals such as transgender persons also presents as an important conduit to better understanding the complex biological features of autoimmunity.

More insights are required to assess how sex and gender may affect the safety, tolerability, and, most importantly, the effectiveness of medications. Research funding in this field can influence public health policies by highlighting the need for sex-specific research and healthcare approaches, which would likely lead to better resource allocation and awareness campaigns to address the unique needs of women in managing AiDs. Additionally, medical education can incorporate these findings to better prepare healthcare professionals in recognizing and treating autoimmune diseases with a sex-specific perspective, in turn helping to improve diagnostic accuracy and tailored patient care.

## Conclusion

The interplay between host and microbe, as well as human vs. mouse-derived data, reveals a highly intricate landscape of sexual dimorphism in autoimmune disorders. Numerous factors, including genetic predispositions, sex hormones, host characteristics, dietary differences, environmental factors, and the gut microbiome, contribute variably across different socioeconomic contexts, converging to influence health outcomes for everyone. The specific impacts of each of these factors and their contribution to the development of AiDs remain poorly elucidated in humans and are highly reliant on animal-derived data, halting the development of effective therapies to treat AiDs (McGee and Huttenhower 2021). Given the clear sex differences in immune responses and in the prevalence/progression of many AiDs, the paucity of sex-based biological differences in biomedical research is no longer acceptable. The importance of studying disease models for both sexes is becoming more evident with research funding bodies, including the National Institutes of Health, now requiring the inclusion of sex as a biological variable in grant applications (Clayton and Collins 2014). Grasping these mechanisms is essential for understanding the pathogenesis of AiDs and suggests the need for sex-specific treatment strategies to optimize efficacy, although systematic studies on treatment responses in males versus females are still limited.

## Data Availability

All source data for this work (or generated in this study) are available upon reasonable request.
